# Efficacy and Safety of Zonisamide Addition in Children With Developmental Epileptic Encephalopathy/Epileptic Encephalopathy: A Real‐World Study

**DOI:** 10.1002/cns.70865

**Published:** 2026-04-03

**Authors:** Peijiao Liu, Li Zhang, Qiao Zeng, XiaoYu Zhao, Li Jiang, Yue Hu

**Affiliations:** ^1^ Department of Neurology Children's Hospital of Chongqing Medical University Chongqing China; ^2^ National Clinical Research Center for Children and Adolescents' Health and Diseases Chongqing China; ^3^ Ministry of Education Key Laboratory of Child Development and Disorders Chongqing China; ^4^ Chongqing Key Laboratory of Child Neurodevelopment and Cognitive Disorders Chongqing China; ^5^ Department of Pediatrics (Fourth Division of General Pediatrics) Yibin Hospital Affiliated to Children's Hospital of Chongqing Medical University Yibin Sichuan China

**Keywords:** add‐on therapy, children, DEE/EE, real‐world study, zonisamide

## Abstract

**Objective:**

To evaluate the real‐world effectiveness and safety of adjunctive zonisamide in children with developmental epileptic encephalopathy/epileptic encephalopathy (DEE/EE), focusing on younger children and highly drug‐resistant cases.

**Methods:**

This open‐label, nonrandomized, self‐controlled, real‐world study included 127 children with DEE/EE at a single center from 2020 to 2025. The primary endpoint was the responder rate (≥ 50% seizure reduction) at 3, 6, 9, and 12 months, analyzed using last observation carried forward. Secondary endpoints included seizure freedom, retention, and adverse events (AEs). Multivariable analysis identified predictors of efficacy.

**Results:**

Responder rates were 51.2%, 55.3%, 53.7%, and 53.7% at 3, 6, 9, and 12 months, respectively. Seizure‐free rates were 23.6%, 26.8%, 24.4%, and 27.6%. Efficacy was consistent across age and etiology. Female gender (OR = 3.00) and fewer prior anti‐seizure medications (OR = 0.77) independently predicted 12‐month response. Among children failing ≥ 5 prior medications, 44.3% responded, and 20.0% achieved seizure freedom. The 12‐month retention rate was 81.9%. AEs occurred in 14.2%, most commonly reduced appetite (7.9%); most were tolerable.

**Conclusion:**

Adjunctive zonisamide provides sustained efficacy and favorable tolerability in children with DEE/EE, including young children < 6 years and highly drug‐resistant cases. Gender and prior treatment history predict long‐term efficacy, supporting its reliable risk–benefit profile for refractory epilepsy.

## Graphical abstract

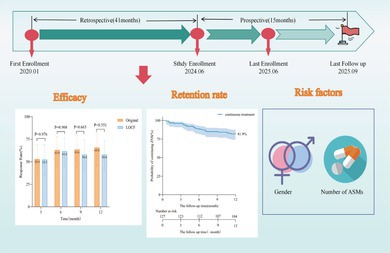

## 1 Introduction

Developmental and epileptic encephalopathy (DEE) is a concept introduced by the International League Against Epilepsy (ILAE) in 2017 to further delineate and distinguish it from epileptic encephalopathy (EE). EE involves severe developmental, cognitive, and behavioral impairments due to frequent epileptiform discharges. Development is typically normal at seizure onset, with delays emerging later. DEE refers to cases where developmental impairment precedes epilepsy. Here, both the underlying etiology and frequent epileptiform discharges contribute to progressive brain dysfunction. Even with full seizure control, encephalopathic features may persist and worsen over time. This combined influence of developmental and epileptic factors is observed across all ages [1]. The overall incidence of DEE is 0.27–0.54 per 1000 newborns, with a mortality rate ranging from 17% to 50%. For neonatal‐onset DEE, mortality exceeds 50% by 2 years of age [2, 3]. Anti‐seizure medications (ASMs) are the foundation of treatment for epileptic encephalopathy. Most patients with DEE/EE are drug‐resistant, and combination therapy with two or more ASMs is often recommended. For those with poor therapeutic response, switching to alternative ASMs (especially newer agents) and evaluating other treatment options are important considerations [4]. Patients with drug‐resistant epilepsy (DRE) face not only elevated risks of accidental injury (approximately 33%) and sudden unexpected death in epilepsy (approximately 1%) [5], but are also strongly associated with adverse neurodevelopmental outcomes and cognitive dysfunction in children [6]. The cumulative incidence of DRE among children with epilepsy is approximately 25% [7]. Identifying effective and well‐tolerated treatment strategies for pediatric patients with DEE/EE is therefore of significant clinical importance.

Zonisamide (ZNS) is a broad‐spectrum, third‐generation anti‐seizure medication with a benzisoxazole‐derived chemical structure. It exhibits multiple mechanisms of action, including blockade of voltage‐gated sodium channels, inhibition of T‐type calcium channels, neuronal membrane stabilization, carbonic anhydrase inhibition, modulation of dopaminergic and serotonergic neurotransmission, antagonism of glutamate excitotoxicity, and free radical scavenging. Animal studies suggest that ZNS modulates glycine receptors to enhance control of neuronal excitability in specific brain regions [8]. Pharmacokinetically, ZNS is rapidly and completely absorbed following oral administration, with a bioavailability of ≥ 90% and a half‐life of 50–70 h, and steady‐state plasma concentrations are typically achieved within approximately 2 weeks. The drug is primarily metabolized by CYP3A4 and should be used with caution in patients with hepatic or renal impairment [9]. Since its initial approval in Japan in 1989, ZNS has been used globally as monotherapy or adjunctive therapy for partial, generalized, and mixed seizures. It is also approved in the European Union as adjunctive treatment for partial epilepsy in children aged ≥ 6 years [10]. In 2009, ZNS was approved by China's National Medical Products Administration (NMPA) as adjunctive therapy for focal seizures in patients aged ≥ 16 years. Clinical studies have demonstrated the efficacy and favorable tolerability of ZNS in adults and older children (≥ 12 years) [11]. However, ZNS use in young children, particularly those under 6 years of age, remains off‐label. Evidence from clinical studies supports its efficacy as adjunctive therapy for seizure control in this population, with reported use in infants as young as 0.96 months [11]. These findings suggest that ZNS could serve as an effective adjunctive therapy for young children (≤ 6 years) with DRE. However, its clinical application requires further validation and optimization through additional high‐quality real‐world studies.

This study enrolled 127 children with DEE/EE, aged 2.1–198.9 months, who received ZNS add‐on therapy at Children's Hospital of Chongqing Medical University between January 1, 2020 and September 1, 2025. A nonrandomized, open‐label, self‐controlled real‐world design was adopted, aiming to evaluate the efficacy and safety of ZNS adjunctive therapy in children with DRE. Stratified analyses by age and epilepsy syndrome type were conducted to explore factors associated with treatment response, thereby providing evidence‐based guidance for the rational use of ZNS in this population.

## 2 Methods

### 2.1 Study Population

#### 2.1.1 Inclusion Criteria

1. Enrollment period: January 1, 2020 to September 1, 2025; Age range: 1 month–18 years.
2. Diagnosis meeting the ILAE 2017 criteria for DRE and DEE/EE [12]; seizure types and epilepsy syndromes classified according to the latest ILAE guidelines [1, 13].
3. ZNS used as adjunctive therapy.

#### 2.1.2 Exclusion Criteria

Patients receiving monotherapy, those with poor compliance, or those for whom an accurate medical history could not be obtained.

### 2.2 Study Methodology

ZNS (manufactured by Shenzhen Zifu Pharmaceutical Co. Ltd., registration certificate no. H20090252) was administered as adjunctive therapy without modifying or replacing the patient's existing ASMs regimen. The initial dose was uniformly set at 2–4 mg/kg/day. Titration schedules and maintenance doses were then individualized according to clinical response and tolerability. The long‐term maintenance doses were categorized as follows: low dose (2–4 mg/kg/day), medium dose (4–8 mg/kg/day), high dose (8–12 mg/kg/day), and very high dose (> 12 mg/kg/day) [14]. The use of ZNS in children under 6 years remains off‐label. In this study, 6 years was selected as the cutoff for age stratification.

Efficacy assessment: The baseline seizure frequency was defined as the mean monthly seizure count during the 3 months prior to ZNS treatment. The seizure reduction rate was calculated as: (baseline frequency − posttreatment frequency)/baseline frequency × 100%. Treatment response was defined as a ≥ 50% reduction in seizure frequency [11].

**FIGURE 1:**
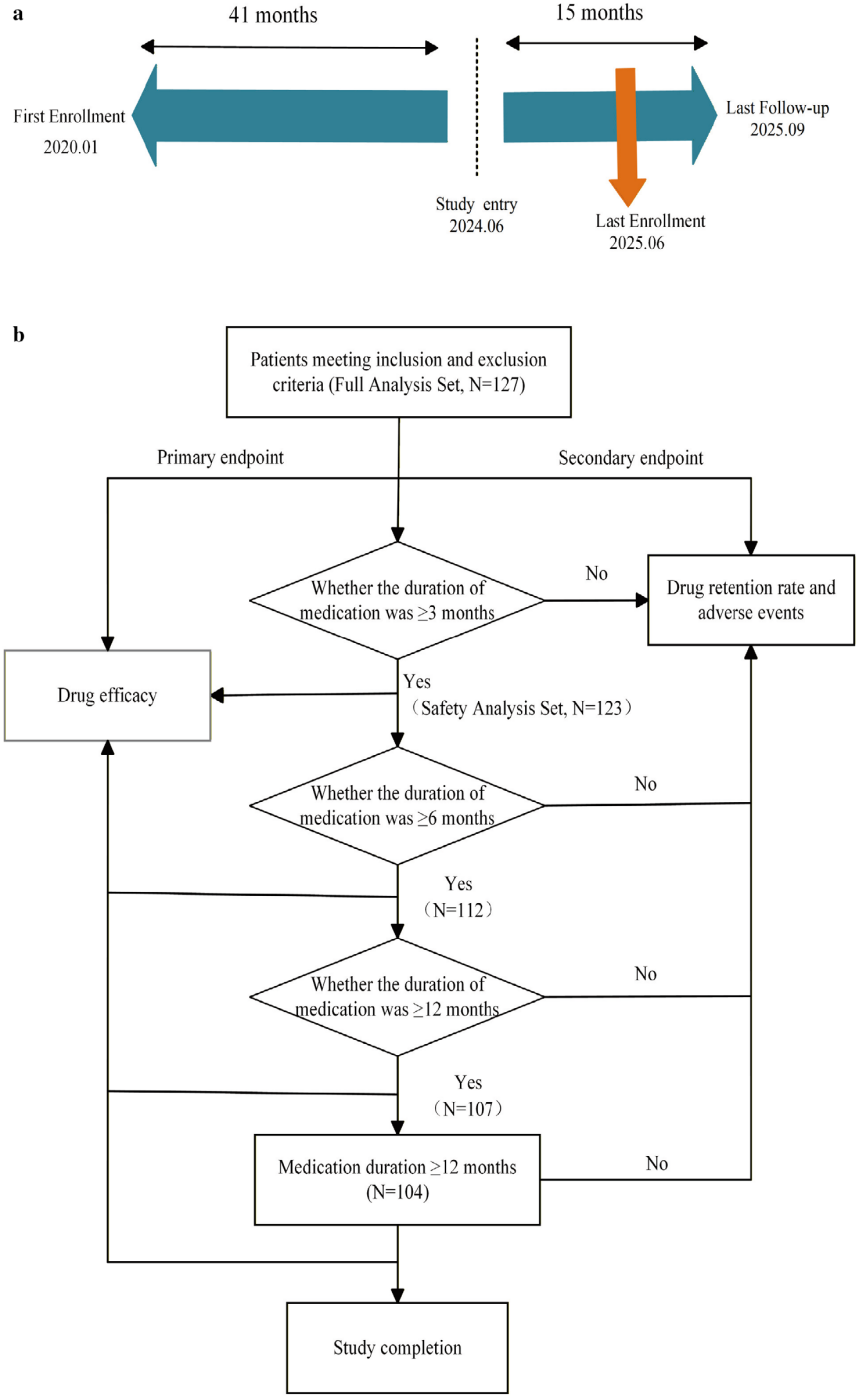
Study design and patient disposition (2020–2025). (a) Study design timeline (2020–2025). The timeline illustrates the study design, including the retrospective phase (41 months) and prospective phase (15 months), enrollment time points (January 2020 and June 2024), and follow‐up cutoff date (September 2025). (b) Patient enrollment and follow‐up flowchart. Of the 127 enrolled children, patients were sequentially screened based on treatment duration (≥ 3, 6, 9, and 12 months). A total of 104 patients completed the 12‐month follow‐up.

This study consisted of a retrospective data collection phase (41 months) followed by a prospective follow‐up phase (15 months; Figure 1a). The primary endpoint was the responder rate for seizure frequency reduction ≥ 50% from baseline (including seizure‐free patients) at 3, 6, 9, and 12 months of ZNS treatment. Secondary endpoints included seizure‐free rates throughout the study period, medication retention rates, and adverse event incidence. Participants who added other treatments (including ketogenic diet, epilepsy surgery, vagus nerve stimulation) or switched antiepileptic drugs during follow‐up were excluded from efficacy assessments but remained in safety evaluations.

Two analysis (Figure 1b) sets were used in this study: (1) Full analysis set: all children who received at least one dose of ZNS and completed ≥ 1 post‐baseline efficacy assessment, for all efficacy analyses. (2) Safety analysis set: all children who received at least one dose of ZNS, for evaluating safety outcomes including adverse events and drug retention rate.

**FIGURE 2:**
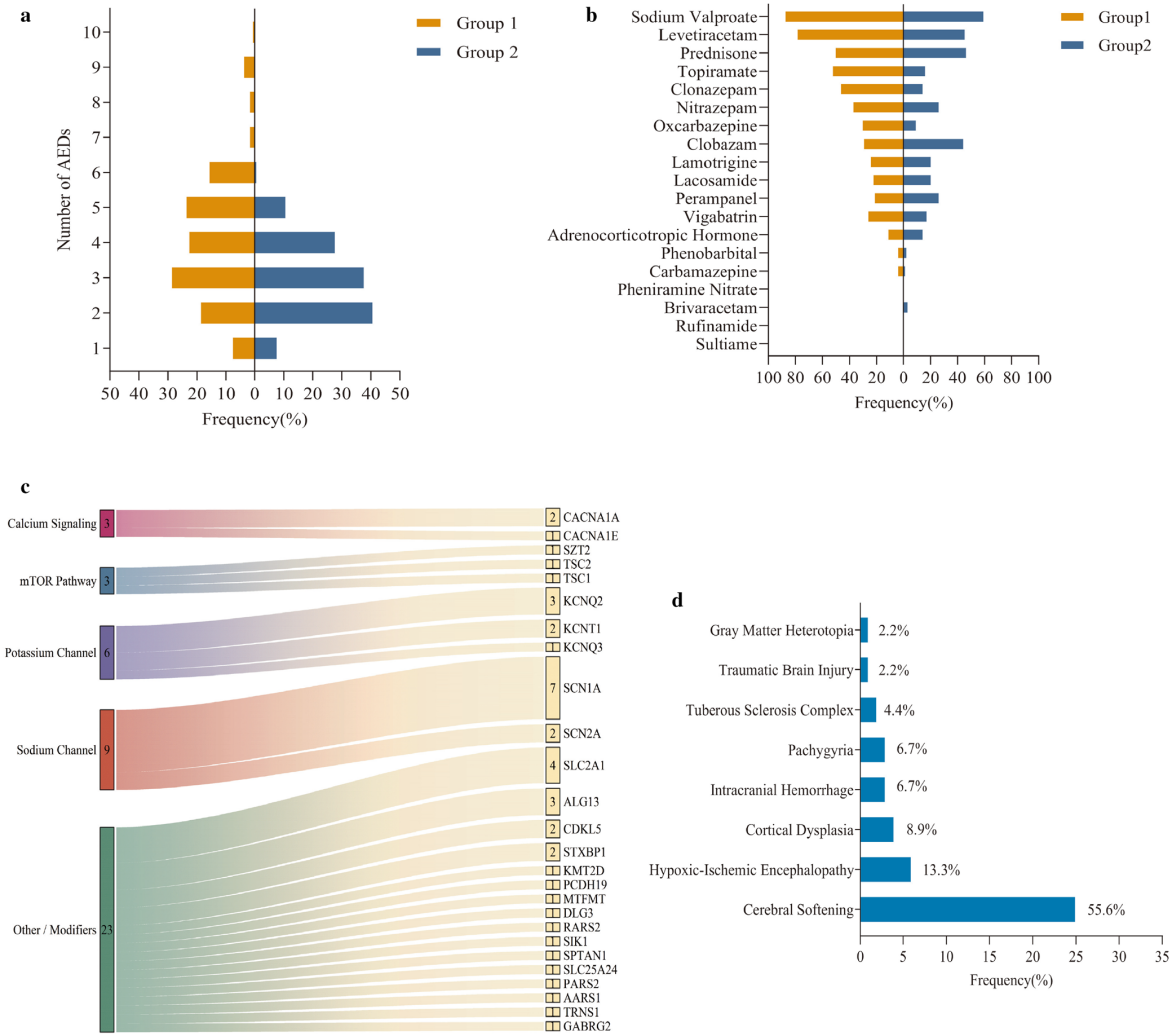
Baseline Clinical Characteristics of the Study Cohort. (a) Comparison of anti‐seizure medication (ASM) types. Symmetrical bar chart showing the distribution of previously used ASMs (Group 1, orange bars) versus ASMs at zonisamide (ZNS) initiation (Group 2, blue bars). The y‐axis lists ASM types, and the x‐axis represents the number of patients. (b) Comparison of number of ASMs used. Symmetrical histogram comparing the number of previously used ASMs (Group 1, orange bars) versus ASMs at ZNS initiation (Group 2, blue bars). The x‐axis represents the number of patients, and the y‐axis represents the number of ASMs (range 1–10). (c) Distribution of hereditary etiologies. Bar chart showing category composition and case numbers of hereditary etiologies in the study cohort. (d) Distribution of structural etiologies. Bar chart showing category composition and case numbers of structural etiologies in the study cohort.

This study employed a self‐controlled before‐after design, using each child's mean seizure frequency during the 3 months prior to ZNS initiation as the baseline to minimize the impact of interindividual heterogeneity on efficacy evaluation. However, as an observational study, this design could not fully exclude the influence of time‐varying confounding factors (e.g., natural disease course, unplanned adjustments of concomitant therapies). Such time‐varying bias was partially controlled by incorporating time‐by‐dose‐adjusted interaction terms in the model and applying Generalized Estimating Equations (GEE) to account for intraindividual correlation, with sensitivity analysis performed to enhance the robustness of the findings.

### 2.3 Statistical Analysis

Statistical analysis was performed using the R software (version 4.4.3). Continuous variables are expressed as median (interquartile range, IQR) or mean ± standard deviation; categorical variables are presented as frequency (percentage).

Statistical analysis accounted for nonrandom missing data arising from treatment discontinuation, loss to follow‐up, or regimen changes by applying the last observation carried forward (LOCF) imputation method. This method may overestimate long‐term efficacy; GEE was used for sensitivity analysis to assess differences before and after imputation. To verify the robustness of the missing data handling, within‐subject correlation was controlled using a Toeplitz covariance structure. Using the imputed dataset, a generalized linear mixed model (GLMM) incorporating time‐by‐dose‐adjusted interaction terms was fitted. The statistical significance of interaction effects was evaluated using the likelihood ratio test (LRT). Baseline variables with *p* < 0.1 in univariate logistic regression were entered into a multivariate logistic regression model to assess their independent effects on treatment response, using a two‐sided *p* < 0.05 as the significance threshold. Multicollinearity was examined using variance inflation factor (VIF). Between‐group comparisons of response rates (age and etiology subgroups) were conducted with chi‐square or Fisher's exact test, followed by the Bonferroni correction; *p* < 0.05 was considered statistically significant. Medication retention was analyzed with Kaplan–Meier survival analysis.

## 3 Results

### 3.1 Baseline Clinical Characteristics

This study included 127 pediatric patients with DEE/EE who received adjunctive ZNS therapy at Children's Hospital of Chongqing Medical University between January 1, 2020, and September 1, 2025 (Table 1, Figure 2). Patient age ranged from 2.1 to 198.9 months. All children who received at least one dose of ZNS (*n* = 127) were included in the safety analysis. Among them, four were excluded from the primary efficacy analysis due to treatment duration < 3 months precluding three‐month efficacy assessment, resulting in an efficacy analysis population of 123 patients.

**TABLE 1:** Baseline characteristics of the study population and univariate analysis of 12‐month treatment response.

Characteristic	Total number, *n* = 127	< 6 years *N* = 93	≥ 6 years, *N* = 34	12 months, OR	*p*
Gender, female	57 (44.9%)	44 (47.3%)	13 (38.2%)	2.75 (1.33–5.80)	0.007
Age at onset of epilepsy, months	21.2 ± 24.0	15.1 ± 15.7	37.6 ± 33.4	1.01 (1.00–1.03)	0.146
Duration of epilepsy before ZNS, months	32.3 ± 40.1	15.1 ± 16.1	79.3 ± 48.1	1.00 (0.99–1.01)	0.595
ZNS‐supplemented treatment duration, months	53.4 ± 45.0	30.2 ± 19.3	117.0 ± 31.7	1.00 (0.99–1.01)	0.734
Family history of epilepsy	9 (7.1%)	6 (6.5%)	3 (8.8%)	1.09 (0.27–4.58)	0.906
Perinatal risk factors	45 (35.4%)	31 (33.3%)	14 (41.2%)	1.22 (0.58–2.58)	0.600
Seizure type					
Focal seizures	56 (44.1%)	38 (40.9%)	18 (52.9%)	1.31 (0.64–2.70)	0.461
Generalized seizures	84 (66.1%)	63 (67.7%)	21 (61.8%)	0.70 (0.32–1.48)	0.348
Generalized tonic‐clonic seizures	19 (15.0%)	10 (10.8%)	9 (26.5%)	0.95 (0.36–2.58)	0.922
Typical absence seizures	3 (2.4%)	2 (2.2%)	1 (2.9%)	—	—
Atypical absence seizures	6 (4.7%)	4 (4.3%)	2 (5.9%)	1.77 (0.33–13.15)	0.517
Myoclonic absence	1 (0.8%)	0	1 (2.9%)	—	—
Myoclonic seizures	17 (13.4%)	10 (10.8%)	7 (20.6%)	0.84 (0.29–2.46)	0.753
Atonic seizures	5 (3.9%)	3 (3.2%)	2 (5.9%)	3.61 (0.52–71.81)	0.257
Tonic seizures	12 (9.4%)	8 (8.6%)	4 (11.8%)	1.83 (0.54–7.17)	0.347
Epileptic spasms	65 (51.2%)	57 (61.3%)	8 (23.5%)	0.46 (0.22–0.95)	0.037
Unknown focal/generalized seizures	4 (3.1%)	2 (2.2%)	2 (5.9%)	0.86 (0.10–7.35)	0.882
DEEs	118 (92.9%)	89 (95.7%)	29 (85.3%)	0.56 (0.11–2.21)	0.422
IESS	44 (34.6%)	41 (44.1%)	3 (8.8%)	0.55 (0.26–1.18)	0.127
LGS	11 (8.7%)	7 (7.5%)	4 (11.8%)	1.04 (0.30–3.80)	0.951
DS	5 (3.9%)	4 (4.3%)	1 (2.9%)	3.61 (0.52–71.81)	0.257
HHE	1 (0.8%)	1 (1.1%)	0	—	—
PME	1 (0.8%)	1 (1.1%)	0	—	—
PCDH19 clustering epilepsy	1 (0.8%)	0	1 (2.9%)	—	—
EE/DEE‐SWAS	2 (1.6%)	2 (2.2%)	0	—	—
EIMFS	1 (0.8%)	1 (1.1%)	0	—	—
EIDEE	6 (4.7%)	6 (6.5%)	0	1.77 (0.33–13.15)	0.517
STXBP1 gene‐associated developmental epilepsy encephalopathy	1 (0.8%)	1 (1.1%)	0	—	—
GLUT1DS	1 (0.8%)	1 (1.1%)	0	—	—
CDKKL5 syndrome	1 (0.8%)	1 (1.1%)	0		—
EE	9 (7.1%)	4 (4.3%)	5 (14.7%)		
Etiology					
Structural	44 (34.6%)	32 (34.4%)	12 (35.3%)	1.06 (0.50–2.23)	0.883
Hereditary	37 (29.1%)	33 (35.5%)	4 (11.8%)	1.04 (0.47–2.29)	0.930
Metabolic	1 (0.8%)	1 (1.1%)	0	—	—
Unknown	45 (35.4%)	27 (29.0%)	18 (52.9%)	0.86 (0.41–1.81)	0.684
Cranial MRI					
Unifocal	8 (6.3%)	4 (4.3%)	4 (11.8%)	1.48 (0.35–7.46)	0.606
Multifocal	31 (24.4%)	23 (24.7%)	8 (23.5%)	1.17 (0.51–2.73)	0.704
Diffuse	30 (23.6%)	21 (22.6%)	9 (26.5%)	0.58 (0.25–1.32)	0.195
Normal	58 (45.7%)	45 (48.4%)	13 (38.2%)	1.22 (0.60–2.50)	0.589
Electroencephalographic discharges					
Focal discharges	56 (44.1%)	36 (38.7%)	20 (58.8%)	1.71 (0.84–3.56)	0.144
Multifocal discharges	18 (14.2%)	13 (14.0%)	5 (14.7%)	0.65 (0.23–1.77)	0.399
Extensive discharge	53 (41.7%)	44 (47.3%)	9 (26.5%)	0.73 (0.35–1.49)	0.386
Background deceleration	92 (72.5%)	69 (74.2%)	23 (67.6%)	0.75 (0.33–1.66)	0.478
ESES	10 (7.9%)	4 (4.3%)	6 (17.6%)	3.26 (0.75–22.54)	0.151
Comorbidities					
Global developmental delay	118 (92.9%)	88 (94.6%)	30 (88.2%)	0.92 (0.22–3.65)	0.906
ADHD	2 (1.6%)	1 (1.1%)	1 (2.9%)	0.86 (0.03–22.12)	0.917
ASD	2 (1.6%)	2 (2.2%)	0	—	—
Language developmental delay	4 (3.1%)	3 (3.2%)	1 (2.9%)	0.86 (0.10–7.35)	0.882
Hemiplegia	3 (2.4%)	3 (3.2%)	0	—	—
Cerebral palsy	10 (7.9%)	6 (6.5%)	4 (11.8%)	1.33 (0.36–5.42)	0.676
Status epilepticus	22 (17.3%)	14 (15.1%)	8 (23.5%)	1.07 (0.41–2.85)	0.895
Treatment					
Number of prior antiepileptic drug treatments	4 (3, 5)	4 (2,5)	4 (3,6)	0.74 (0.59–0.91)	0.007
Number of combined ASMs treatments	3 (2, 4)	3 (2,4)	3 (2,3)	0.73 (0.51–1.02)	0.073
Hormone pulse therapy	77 (60.6%)	61 (65.6%)	16 (47.1%)	1.19 (0.58–2.47)	0.633
Ketogenic diet therapy	39 (30.7%)	35 (37.6%)	4 (11.8%)	0.40 (0.18–0.87)	0.023
Combined VNS therapy	5 (3.9%)	3 (3.2%)	2 (5.9%)	3.61 (0.52–71.81)	0.257
Surgery	5 (3.9%)	5 (5.4%)	0	3.61 (0.52–71.81)	0.257
Adverse reactions	18 (14.2%)	10 (10.8%)	8 (23.5%)	1.31 (0.64–2.70)	0.461
Hepatorenal dysfunction	1 (0.8%)	0	1 (2.9%)	—	—
Rash	1 (0.8%)	1 (1.1%)	0	—	—
Reduced appetite	10 (7.9%)	5 (5.4%)	5 (14.7%)	0.40 (0.08–1.61)	0.217
Nephrolithiasis	1 (0.8%)	1 (1.1%)	0		
Irritability	2 (1.6%)	1 (1.1%)	1 (2.9%)	0.86 (0.03–22.12)	0.917
Somnolence	1 (0.8%)	0	1 (2.9%)	—	—
Hypohidrosis	2 (1.6%)	1 (1.1%)	1 (2.9%)	—	—
Drug‐induced fever	1 (0.8%)	1 (1.1%)	0	—	—
Anemia	1 (0.8%)	1 (1.0%)	0	—	—
Discontinued due to adverse reactions	4 (3.1%)	3 (3.2%)	1 (2.9%)	—	—
Maintenance dose					
Low dose	15 (11.8%)	6 (6.5%)	9 (26.5%)	1.17 (0.38–3.77)	0.781
Medium dose	47 (37.0%)	28 (30.1%)	19 (55.9%)	1.41 (0.67–3.00)	0.368
High dose	48 (37.8%)	43 (46.2%)	5 (14.7%)	0.90 (0.44–1.87)	0.779
Very high dose	17 (13.4%)	16 (17.2%)	1 (2.9%)	0.56 (0.19–1.56)	0.271
Dose adjustment during treatment	37 (29.1%)	28 (30.1%)	9 (26.5%)	0.95 (0.44–2.08)	0.900

*Note:* Continuous variables are expressed as mean ± standard deviation or median (interquartile range). Categorical variables are presented as *n* (%). No missing values; data exclusion: Four pediatric patients received medication for less than 3 months. The left section of this table presents baseline characteristics for the overall cohort (*N* = 127) and by age subgroup. The two right columns show results from univariate logistic regression analysis in 123 patients, with ≥ 50% response rate at 12 months as the dependent variable. Age stratification was descriptive only.

Abbreviations: ADHD, attention deficit hyperactivity disorder; ASD, autism spectrum disorder; ASMs, anti‐seizure medications; CDKL5 syndrome, CDKL5‐developmental epileptic encephalopathy; CNS, central nervous system; DEE, developmental epileptic encephalopathy; DEE/EE‐SWAS, developmental epileptic encephalopathy/epilepsy with spastic waddling and ataxia syndrome (epilepsy with sleep‐related spike‐and‐wave); DS, Dravet Syndrome; EE, epileptic encephalopathy; EEG, electroencephalogram; EIDEE, early‐onset infantile developmental epileptic encephalopathy; EIMFS, infantile epilepsy with migrating focal seizures; GLUT1DS, glucose transporter 1 deficiency syndrome; HHE, hemiplegic epilepsy with hemiparesis; IESS, infantile epileptic spasm syndrome; LGS, Lennox–Gastaut syndrome; MRI, magnetic resonance imaging; PME, progressive myoclonic epilepsy; VNS, vagus nerve stimulation; ZNS, zonisamide.

### 3.2 Efficacy Analysis

**FIGURE 3:**
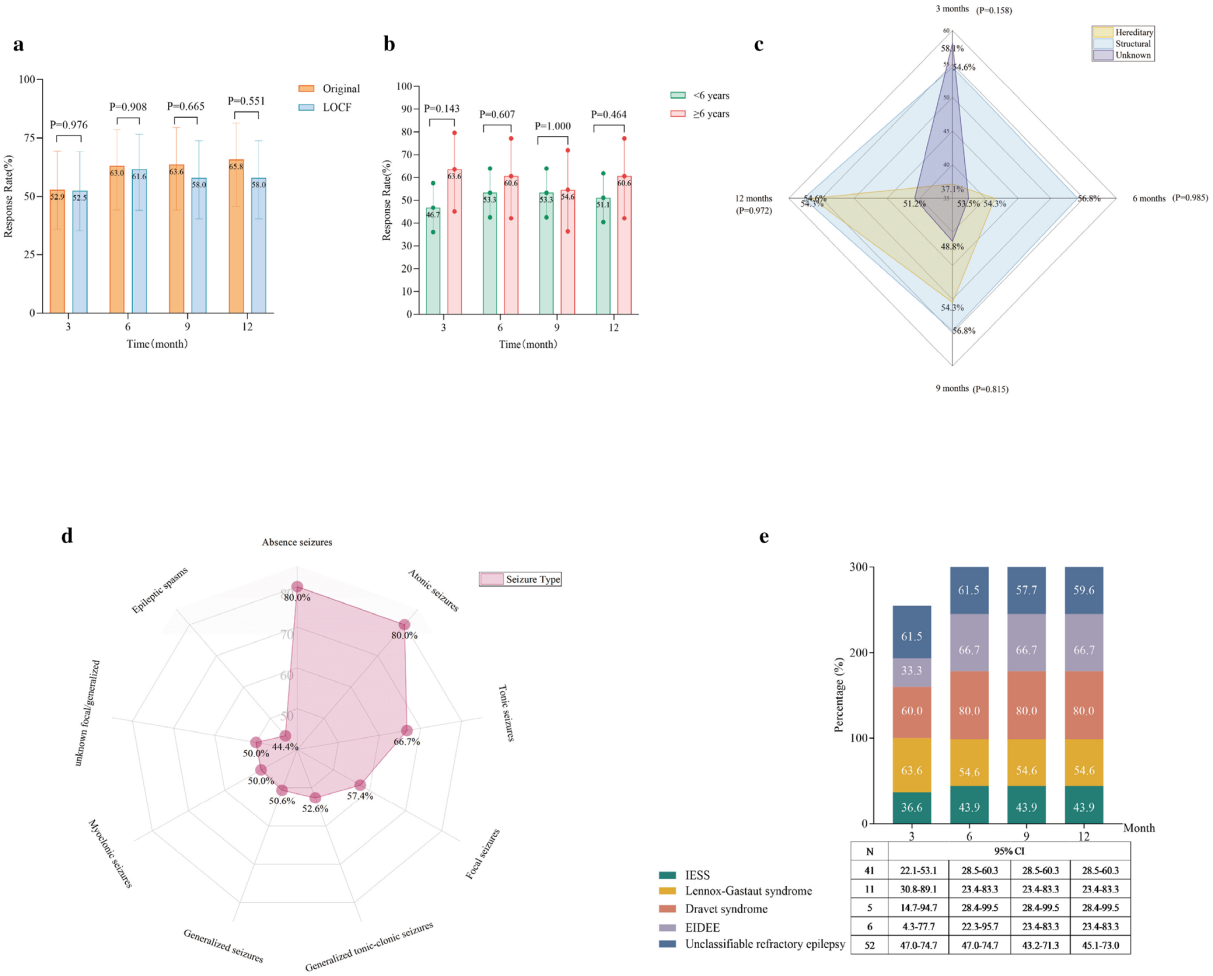
Comparison of ≥ 50% response rate between raw data and LOCF imputation at different follow‐up time points, and subgroup efficacy analyses of zonisamide add‐on treatment. (a) Comparison of ≥ 50% response rate between raw data and LOCF imputation. Orange bars represent responder rates based on observed data from evaluable patients; blue bars represent rates after LOCF imputation for missing data due to discontinuation or loss to follow‐up. Sensitivity analysis assessed the impact of missing data on primary conclusions. *p*‐values above each time point indicate comparisons between raw and imputed data. No statistically significant differences were observed at any time point (all *p* > 0.05), suggesting robustness of the primary efficacy findings to missing data handling. (b) ≥ 50% response rate by age group (< 6 years vs. ≥ 6 years). Patients were stratified into < 6 years (*n* = 93) and ≥ 6 years (*n* = 34). Bar charts show responder rates at 3, 6, 9, and 12 months post‐ZNS initiation. Error bars indicate 95% confidence intervals. Between‐group comparisons at each time point were performed using chi‐square tests, with *p*‐values shown. No significant differences were observed at any time point (all *p* > 0.05), indicating comparable efficacy in younger children. (c) ≥ 50% response rate by etiology. Radar chart comparing responder rates at 3, 6, 9, and 12 months across three etiological categories: Hereditary (yellow), structural (blue), and unknown (purple). Each axis represents a follow‐up time point, with filled area size reflecting the responder rate for each etiology. (d) ≥ 50% response rate by seizure type at 12 months. Radar chart showing responder rates across various seizure types. Uniform red filled areas represent data for all seizure types. Each axis corresponds to a specific seizure type, with scale percentages indicating responder rates. (e) ≥ 50% response rate by epilepsy syndrome. Combined bar chart and data table. Left panel shows responder rates at 3, 6, 9, and 12 months for five specific epilepsy syndromes. Right panel lists sample sizes (N) and 95% confidence intervals for responder rates at key time points (e.g., 12 months).

A total of 123 patients were included in the efficacy analysis. Compared with baseline, the mean reduction in seizure frequency with adjunctive ZNS at 3, 6, 9, and 12 months was 40.0%, 46.3%, 43.3%, and 37.0%, respectively (all *p* < 0.001), demonstrating sustained efficacy in reducing seizure burden. LOCF imputation was applied in 27 patients. Sensitivity analysis comparing pre‐ and post‐imputation data revealed no statistically significant differences in efficacy at any follow‐up time point (Figure 3a). Using the imputed dataset, the responder rates (≥ 50% reduction) at 3, 6, 9, and 12 months of ZNS treatment were 51.2% (63/123), 55.3% (68/123), 53.7% (66/123), and 53.7% (66/123), respectively. Corresponding seizure‐free rates were 23.6% (29/123), 26.8% (33/123), 24.4% (30/123), and 27.6% (34/123). GLMM analysis showed that dose adjustment (whether performed or not) did not significantly affect efficacy at any time point (all *p* > 0.05), indicating no significant interaction between dose adjustment and time.

Patients were stratified by age into two groups: ≥ 6 years (*n* = 34) and < 6 years (*n* = 93) for comparative analysis. Efficacy did not differ significantly between these age groups at any follow‐up time point (Figure 3b). Furthermore, ZNS showed comparable response rates across different etiological subgroups, with no significant differences observed at 3, 6, 9, or 12 months (Figure 3c). Notably, the 12‐month response rate reached 85.7% in patients with SCN1A‐related epilepsy. At 12 months, the ≥ 50% response rates were 57.4% (31/54) for focal seizures and 50.6% (41/81) for generalized seizures. Among generalized seizure subtypes, the 12‐month response rates were 44.4% (28/63) for clonic, 50.0% (8/16) for myoclonic, 80.0% (4/5) for atonic, and 80.0% (8/10) for absence seizures (Figure 3d). For epilepsy syndromes, the 12‐month response rates were: 80% for Dravet syndrome (DS), 66.7% for early infantile developmental and epileptic encephalopathy (EIDEE), 59.6% for unclassified DEE, 54.6% for Lennox–Gastaut syndrome (LGS), and 43.9% for infantile epileptic spasms syndrome (IESS) (Figure 3e).

**FIGURE 4:**
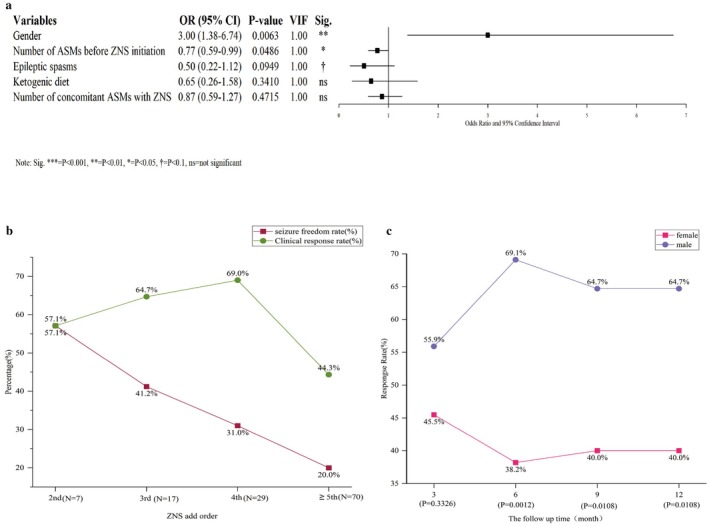
Risk Factors for Efficacy of ZNS Add‐On Therapy in Children with DEE/EE. (a) Multivariate logistic regression forest plot of factors associated with ≥ 50% response rate at 12 months. Forest plot showing results of multivariate logistic regression with ≥ 50% response rate at 12 months as the dependent variable. The y‐axis lists predictor variables assessed in the model, including age, sex, number of concomitant ASMs, specific seizure type/syndrome, and concomitant structural brain lesions. The x‐axis represents odds ratio. For each variable, the central square indicates the point estimate and the horizontal line represents the 95% confidence interval. Confidence intervals crossing the null line indicate no significant association. (b) ≥ 50% response rate and seizure‐free rate at 12 months by ZNS add‐on sequence. Line chart showing trends in seizure‐free rate and ≥ 50% response rate according to ZNS add‐on sequence (i.e., position in the sequence of ASMs used). The x‐axis represents the number of prior ASMs before ZNS initiation. The left y‐axis and purple diamond line represent seizure‐free rate; the right y‐axis and green square line represent ≥ 50% response rate. Solid points indicate the number of patients with evaluable data at each add‐on sequence. The figure explores differences in cumulative success rates between early addition (e.g., as 2nd–4th ASM) and later addition (e.g., as 5th–7th ASM) in achieving seizure‐free status and ≥ 50% response rate. (c) ≥ 50% response rate by sex at different time points. Line chart comparing treatment response trajectories between male and female children at 3, 6, 9, and 12 months after ZNS add‐on. The x‐axis represents treatment duration and the y‐axis represents responder rate. The solid blue line represents male children and the dotted pink line represents female children.

Using a ≥ 50% reduction in seizure frequency at the 12‐month follow‐up as the outcome variable, univariate logistic regression was performed on baseline characteristics (Table 1). Variables with *p* < 0.1 were entered into a multivariate logistic regression model. VIF for all included variables was below two, indicating no significant multicollinearity and good model stability. Multivariate analysis identified gender (OR = 3.00, 95% CI 1.38–6.74; *p* = 0.0063) and the number of ASMs before ZNS initiation (OR = 0.77, 95% CI 0.59–0.99; *p* = 0.0486) as independent predictors of 12‐month treatment response (Figure 4a). Figure 4b presents the ≥ 50% response rate and seizure‐free rate at 12 months of ZNS treatment by add‐on sequence. Figure 4c shows the ≥ 50% response rate at 3, 6, 9, and 12 months by sex.

### 3.3 Drug Retention Rate and Safety

**FIGURE 5:**
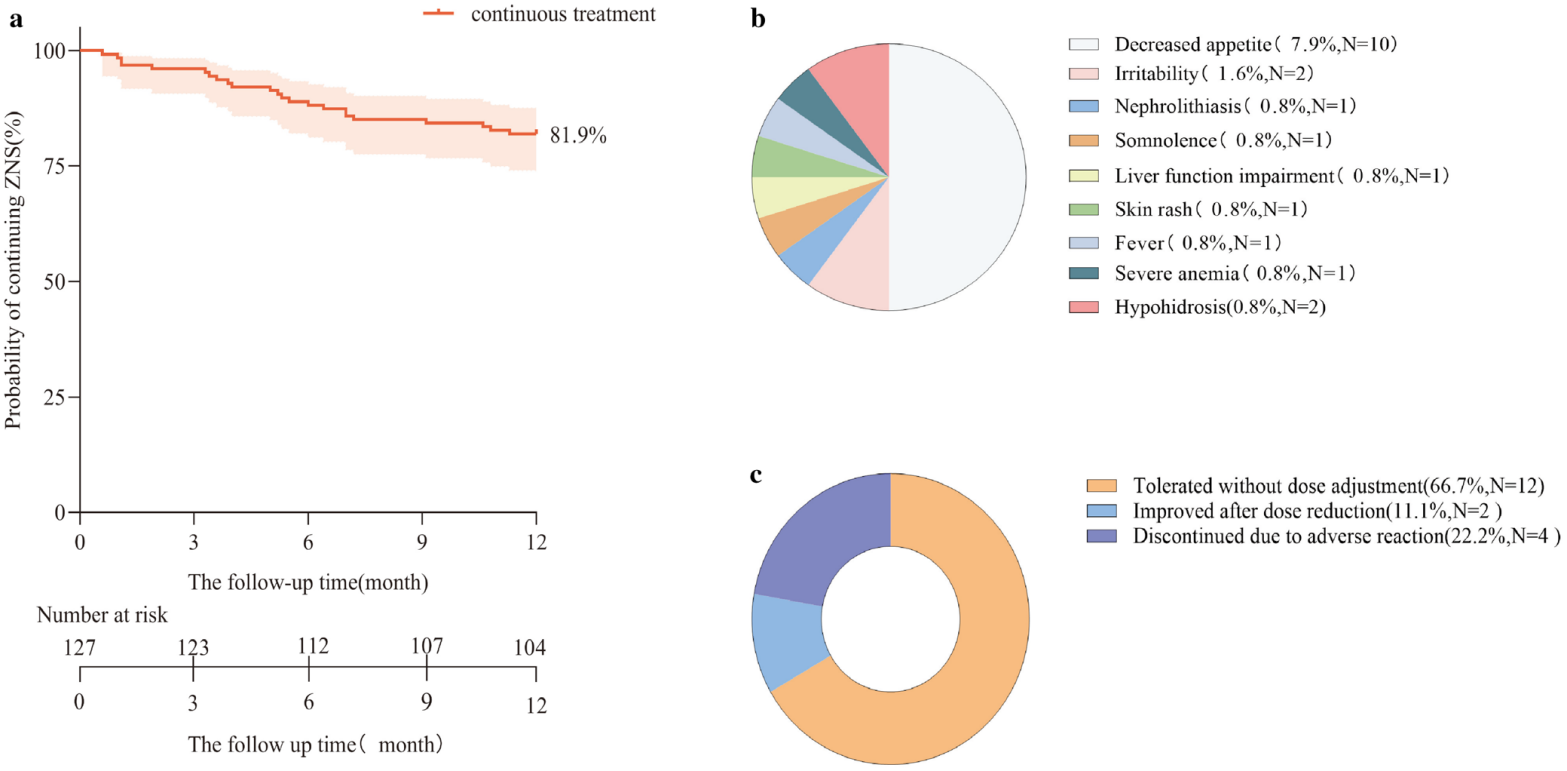
Safety Analysis of ZNS Add‐On Treatment. (a) Twelve‐month drug retention rate of zonisamide add‐on therapy. Kaplan–Meier survival curve showing cumulative probability of continued ZNS treatment in children with DEE/EE over 12 months of follow‐up. The x‐axis represents follow‐up time (months) and the y‐axis represents drug retention rate (%). Vertical tick marks on the curve indicate censored events (e.g., end of follow‐up or loss to follow‐up). The pink line represents the overall patient survival curve. (b) Types and distribution of adverse reactions reported during ZNS treatment. Different color blocks represent different types of adverse reactions. The most common adverse reaction was decreased appetite (7.9%, *N* = 10). Other reactions included irritability and hypohidrosis, all with low incidence rates. (c) Dose adjustment strategies following zonisamide‐related adverse reactions. Quantitative analysis of clinical management patterns for ZNS adverse reactions showed that the majority (66.7%) of children tolerated adverse reactions without requiring dose adjustment.

Among the 127 pediatric patients receiving adjunctive ZNS, the median treatment duration was 12 months (range 0.6–12.0), with a median time to discontinuation of 5.2 months (IQR 3.3–7.0). Kaplan–Meier analysis (Figure 5a) showed retention rates of 96.8% (123/127), 88.2% (112/127), 84.3% (107/127), and 81.9% (104/127) at 3, 6, 9, and 12 months, respectively, indicating favorable treatment persistence. Adverse events were reported in 14.2% (18/127) of patients. Reduced appetite (7.9%, 10/127) was the most common, followed by irritability and hypohidrosis (each 1.6%) (Figure 5b). Most events were mild‐to‐moderate; 66.7% (12/18) of affected patients tolerated treatment without dose adjustment, 11.1% (2/18) improved after dose reduction, and 22.2% (4/18) discontinued due to adverse events, including one case of severe hepatic and renal impairment (Figure 5c).

## 4 Discussion

### 4.1 Efficacy Analysis

This single‐center, exploratory real‐world study comprised all children with DEE/EE who received add‐on ZNS at this center between January 1, 2020 and September 1, 2025, and met all eligibility criteria. The sample size was determined by the actual patient cohort, reflecting routine clinical practice. Compared with baseline, mean seizure frequency decreased by 40.0%, 46.3%, 43.3%, and 37.0% at 3, 6, 9, and 12 months after ZNS addition, respectively (all *p* < 0.001), indicating sustained and effective seizure reduction with add‐on ZNS therapy. The statistically significant differences suggest that the current sample size provided sufficient power to detect differences in the primary endpoint. For children with DRE, previous reports indicate ZNS adjunctive therapy yields response rates of 37.8%–56.5% and seizure‐free rates of 6.6%–15.3% [15, 16, 17, 18], This study demonstrates that adjunctive ZNS consistently reduces seizure frequency in children with DEE/EE, maintaining a response rate above 50% and a seizure‐free rate of approximately 25% across all follow‐up time points. No significant difference in efficacy was observed between children aged ≥ 6 years and those < 6 years, supporting the clinical feasibility of ZNS in younger children with DEE/EE [19].

In the etiological analysis, ZNS showed comparable efficacy among children with structural, genetic, or unknown causes of epilepsy. The underlying etiology of childhood epilepsy is highly heterogeneous. Notably, genetic or presumed genetic causes represent approximately 30% of childhood‐onset epilepsy [20] and up to 80% of infantile‐onset epilepsy [21]. ZNS's multiple mechanisms of action position it as a promising targeted therapeutic agent for genetic epilepsies [22]. It has shown particularly high efficacy in patients with epilepsy related to specific genes such as SCN1A and KCNA2 [23, 24]. In this study, genetic etiologies constituted 29.1% of cases. Adjunctive ZNS yielded a ≥ 50% response rate of 52.3% in children with genetic etiologies, reaching 85.7% in those with SCN1A‐related epilepsy, supporting its potential advantage in specific genetic epilepsies. Post‐marketing observational studies further confirm its broad‐spectrum anti‐seizure activity, including efficacy against generalized tonic‐clonic, absence, and myoclonic seizures [25].

As monotherapy, ZNS achieves response rates of 70%–92.9% for focal seizures and 58%–91.2% for generalized seizures, with seizure‐free rates reaching 63%–79% [19, 26, 27]. As adjunctive therapy, response rates range from 26.9% to 73.6% for focal seizures [16, 27, 28], and from 25% to 56.5% for generalized seizures [16, 19, 29, 30]. In this study, adjunctive ZNS for children with DEE/EE achieved responder rates (≥ 50% reduction) of 57.4% for focal seizures and 50.6% for generalized seizures. As a calcium channel blocker, ZNS primarily inhibits T‐type Ca^2+^ channels without affecting L‐type channels, which may explain its efficacy in absence seizures, with reported responder rates of 46.3%–87.5% and seizure‐free rates of 14.8%–50% [30, 31, 32]. In this study, the responder rate for absence seizures was 80%. The multiple mechanisms of action of ZNS may enhance its potential for complementary use with other anti‐seizure medications, offering a promising therapeutic option for diverse epilepsy syndromes [33]. ZNS demonstrated efficacy across several syndromes, with responder rates of 80% for DS, 66.7% for EIDEE, 59.6% for unclassified DEE, 54.6% for LGS, and 43.9% for IESS. In a previous report, two of six patients with DS showed a ≥ 50% reduction in tonic‐clonic and myoclonic seizures following ZNS treatment. Additionally, ZNS demonstrated favorable prophylactic efficacy in 13.5% of DS patients with status epilepticus [16, 34]. In children with LGS treated with adjunctive ZNS, 4.8% achieved complete seizure control, and 46.8% attained a > 50% reduction in seizure frequency [16]. A Korean multicenter clinical study reported that 33 patients (51.6%) achieved a > 50% reduction in seizure frequency with ZNS. this effect was observed even in typically more refractory seizure types such as atonic and myoclonic seizures [35]. Studies have confirmed that ZNS has rapid onset and definite efficacy in treating [36]. Among children continuing treatment, 20.3% achieved spasm resolution within 2 weeks, and 64% of responders remained spasm‐free by the end of follow‐up [37, 38]. A study from Texas, USA, reported that 26% of children with refractory symptomatic infantile spasms experienced spasm cessation with ZNS, with 13% achieving success on ZNS monotherapy [39]. However, other studies suggest its efficacy against spasms may be limited [40].

In this study, gender and the number of ASMs used prior to ZNS initiation were independent predictors of long‐term efficacy. Multiple studies have established an association between gender and DRE [7, 41, 42]. Patients with DRE exhibit sex‐related differences in peripheral complement system activity and cytokine profiles. Males show broader dysregulation across the classical, lectin, and terminal complement pathways, along with a tendency toward elevated CCL2 and CCL5, whereas females display reduced levels of TNF‐*α* and IL‐8. These sex‐specific immune alterations may influence neuronal excitability, drug metabolism, or target sensitivity, potentially contributing to the observed differences in treatment response [43]. Furthermore, menstrual cycle–associated epilepsy in women is a recognized risk factor for drug‐resistant idiopathic generalized epilepsy, underscoring the complex neuroendocrine‐immune interplay that may underlie sex‐based differences in treatment response. A higher number of ASMs used prior to ZNS initiation was associated with poorer long‐term efficacy, directly reflecting greater disease refractoriness. Previous failure of multiple drugs typically indicates activation of broader resistance mechanisms, including upregulation of multidrug transporters and target modifications. For instance, such mechanisms were reported in 12% of patients who had failed two prior drugs, compared with only 2.5% of those who had failed five or more [44]. When used as a second‐line adjunctive agent in children with DEE/EE, ZNS achieved a 12‐month response rate (≥ 50% seizure reduction) of 57.1% and a seizure‐free rate of 57.1%. However, among children who had previously failed five or more ASMs, the response rate declined to 44.3% and the seizure‐free rate dropped to 20.0%. The proportion of children with DEE/EE achieving seizure freedom through pharmacotherapy progressively decreased as the number of ASMs used before initiating ZNS increased. This does not imply resistance to all medications. Even among DEE/EE children refractory to ≥ 5 ASMs, ZNS demonstrated a 44.3% efficacy rate, indicating therapeutic potential for highly drug‐resistant populations, likely attributable to its multi‐mechanism action. Therefore, regardless of other therapeutic attempts, ASMs remain the fundamental treatment for DEE/EE. Clinically, in addition to actively considering surgical evaluation or other potentially effective non‐pharmacological therapies, it remains important to continue trialing alternative ASMs, with particular attention to newer broad‐spectrum agents.

### 4.2 Drug Retention Rate and Safety Analysis

Drug retention rate is a key indicator for evaluating the long‐term sustainability and tolerability of treatment. In this study, adjunctive ZNS achieved a 12‐month retention rate of 81.9%, which exceeds the 54.4%–75% rates reported in prior cohorts over 12–24 months [16, 45, 46]. These findings support the potential role of ZNS as a viable option for long‐term management in children with epilepsy.

In this study, the overall incidence of treatment‐emergent adverse events (TEAEs) was 14.2%, most commonly decreased appetite (7.9%). The withdrawal rate due to TEAEs was 3.1%, consistent with prior reports [47, 48]. Adverse reactions occur primarily during the initial phase of treatment. These can be effectively mitigated through strategies such as gradual dose titration, low starting doses, and close clinical monitoring, thereby improving treatment adherence and long‐term retention rates in pediatric patients [49].

## 5 Conclusion

This single‐center, open‐label, real‐world study provides the first systematic evaluation of the long‐term efficacy, safety, and predictive factors of ZNS adjunctive therapy in children with DEE/EE, offering key evidence to guide clinical practice. This study focused on young children (< 6 years) and those with highly refractory epilepsy (having used ≥ 5 ASMs), thereby extending the evidence for ZNS use in off‐label age groups and treatment‐resistant scenarios. The real‐world design closely reflects clinical practice and confirms the sustained, broad‐spectrum efficacy of ZNS across different ages, etiologies, and refractory epilepsy syndromes (e.g., Dravet syndrome, LGS, IESS). Multivariate analysis identified gender and the number of prior ASMs as significant predictors of long‐term efficacy, providing a basis for individualized treatment. ZNS demonstrated good tolerability and high retention rates across the overall cohort, maintaining efficacy even in children with multiple treatment failures. Consequently, ZNS represents an effective and safe adjunctive treatment option for children with DEE/EE, particularly younger and drug‐resistant patients. Its efficacy correlates with gender and treatment history, offering significant clinical guidance value.

## 6 Limitations

(1) As a single‐center real‐world study, the findings reflect the diagnostic and treatment practices and patient population of one institution; while the results have some clinical extrapolation, their generalizability requires validation through multicenter studies. The self‐controlled before‐after design, although closer to real‐world clinical practice and suitable for evaluating incremental benefits of add‐on therapy, lacks a randomized control group and may be subject to unmeasured confounding factors. (2) despite the inclusion of 127 patients, the high etiological and syndromic heterogeneity of DEE/EE resulted in limited sample sizes for certain subgroups (e.g., specific rare syndromes), potentially reducing statistical power for subgroup comparisons (e.g., by etiology or syndrome) and limiting in‐depth efficacy analyses in more refined populations. (3) the combination of retrospective and prospective design elements, while reflective of real‐world clinical practice, may have resulted in less complete or standardized data collection during the retrospective phase compared with rigorous prospective trials. (4) LOCF method used to handle nonrandom missing data (e.g., loss to follow‐up) may introduce bias and may not fully reflect outcomes after treatment discontinuation. (5) the maximum follow‐up of 12 months precluded comprehensive assessment of ZNS's potential long‐term effects on cognitive development.

## Author Contributions

**Yue Hu:** conceptualization, writing – review and editing, supervision, resources. **Peijiao Liu:** methodology, visualization, formal analysis, investigation, writing – original draft. **Li Zhang:** investigation. **Qiao Zeng:** methodology. **XiaoYu Zhao:** project administration. **Li Jiang:** resources.

## Funding

This study was funded by the Chongqing Municipal Health Commission (Grant No. 2023jstg032).

## Disclosure

All authors confirm that no generative artificial intelligence (AI) technologies, such as large language models (e.g., ChatGPT), text‐to‐image generators, or automated reasoning tools, were used in any stage of the preparation of this manuscript, including but not limited to: idea generation, literature search, data analysis, writing, editing, or image creation. The authors take full responsibility for the entire content of this work.

## Ethics Statement

This study was approved by the Medical Ethics Committee of Children's Hospital of Chongqing Medical University (Approval No.: 2024 Lun Shen (Lin Yan) No. (381)). Before initiating ZNS therapy, investigators discussed the study objectives, procedures, potential risks, and benefits in detail with the guardians, and written informed consent was obtained. All human studies have been reviewed by the appropriate ethics committee and have therefore been performed in accordance with the ethical standards laid down in an appropriate version of the Declaration of Helsinki (as revised in Brazil 2013). For any use outside the approved indications by China's NMPA, guardians were explicitly informed in the consent form regarding the off‐label nature of treatment, along with a thorough explanation of the expected efficacy and possible adverse reactions, ensuring the adequacy and compliance of the informed consent process.

## Conflicts of Interest

The authors declare no conflicts of interest.

## Data Availability

The data that support the findings of this study are available from the corresponding author upon reasonable request.
